# Additional Value of CH_4_ Measurement in a Combined ^13^C/H_2_ Lactose Malabsorption Breath Test: A Retrospective Analysis

**DOI:** 10.3390/nu7095348

**Published:** 2015-09-07

**Authors:** Els Houben, Vicky De Preter, Jaak Billen, Marc Van Ranst, Kristin Verbeke

**Affiliations:** 1 Translational Research Center for Gastrointestinal Disorders (TARGID), KU Leuven, Leuven 3000, Belgium; E-Mails: Els.Houben@uzleuven.be (E.H.); Vicky.DePreter@ucll.be (V.D.P.); 2 Clinical Department of Laboratory Medicine, University Hospitals Leuven, Leuven 3000, Belgium; E-Mails: Jaak.Billen@uzleuven.be (J.B.); Marc.VanRanst@uzleuven.be (M.V.R.); 3 Group Health and Social Work, University College Leuven-Limburg (UCLL), Leuven 3000, Belgium; 4 Laboratory of Clinical and Epidemiological Virology, KU Leuven, Leuven 3000, Belgium; 5 Leuven Food Science and Nutrition Research Centre (LFoRCe), Leuven 3000, Belgium

**Keywords:** lactose malabsorption, lactase deficiency, methane, hydrogen, breath test, stable isotopes

## Abstract

The lactose hydrogen breath test is a commonly used, non-invasive method for the detection of lactose malabsorption and is based on an abnormal increase in breath hydrogen (H_2_) excretion after an oral dose of lactose. We use a combined ^13^C/H_2_ lactose breath test that measures breath ^13^CO_2_ as a measure of lactose digestion in addition to H_2_ and that has a better sensitivity and specificity than the standard test. The present retrospective study evaluated the results of 1051 ^13^C/H_2_ lactose breath tests to assess the impact on the diagnostic accuracy of measuring breath CH_4_ in addition to H_2_ and ^13^CO_2_. Based on the ^13^C/H_2_ breath test, 314 patients were diagnosed with lactase deficiency, 138 with lactose malabsorption or small bowel bacterial overgrowth (SIBO), and 599 with normal lactose digestion. Additional measurement of CH_4_ further improved the accuracy of the test as 16% subjects with normal lactose digestion and no H_2_-excretion were found to excrete CH_4_. These subjects should have been classified as subjects with lactose malabsorption or SIBO. In conclusion, measuring CH_4_-concentrations has an added value to the ^13^C/H_2_ breath test to identify methanogenic subjects with lactose malabsorption or SIBO.

## 1. Introduction

Lactose malabsorption refers to a condition in which the disaccharide lactose, a carbohydrate exclusively occurring in mammalian milk, is not completely digested in the small intestine and reaches the large intestine. Small intestinal digestion of lactose occurs by the brush border enzyme lactase, also known as lactase-phlorizin hydrolase, which belongs to the family of β-galactosidase enzymes. Lactase hydrolyses lactose into its constituent monosaccharides glucose and galactose that are subsequently absorbed by the intestinal mucosa. Unabsorbed lactose enters the large intestine where it is fermented by the resident microbiota. Major end products include short chain fatty acids (SCFA) comprising acetate, propionate and butyrate as well as gasses such as hydrogen (H_2_), methane (CH_4_) and carbon dioxide (CO_2_).

Humans are generally born with high levels of lactase activity. However, these levels decline after weaning and reach a stable, low level at the age of about 5–10 years [1]. Only in some populations, especially Caucasians, lactase activity persists at high levels at adult age due to a single nucleotide polymorphism (SNP) within an intron upstream of the lactose coding region (13910C/T with the T-variant causing persistence and the C-variant leading to non-persistence) [2]. Besides hypolactasia, gastrointestinal disorders that affect the small intestinal mucosal integrity such as Crohn’s disease, coeliac disease or gut infections [3], or pelvic radiotherapy [4] can also induce lactose malabsorption. This so-called secondary lactose malabsorption disappears upon healing of the mucosa.

In subjects with lactose malabsorption, the intake of lactose can, but does not necessarily, induce gastrointestinal symptoms including nausea, bloating, flatulence, diarrhea, borborygmi and abdominal pain. Besides, also systemic symptoms have been reported such as headache, fatigue or skin disease [5]. Those subjects are considered to be lactose intolerant. Importantly, lactose intolerance is not a synonym to lactose malabsorption but specifically refers to the experience of discomfort after intake of lactose in subjects with hypolactasia. Only one third up to half of the patients with lactose malabsorption is also lactose intolerant [6,7].

Several tests are available to detect lactase deficiency and lactose malabsorption. The most direct approach, which has therefore been proposed as the golden standard, is the assessment of lactase activity in jejunal biopsies [8]. However, this test is invasive and the result may be influenced by the fact that lactase is irregularly expressed in the intestinal mucosa which limits its widespread application [9]. Other tests involve the measurement of the increase in serum glucose after oral lactose administration or genetic testing to detect SNPs in the lactase gene located on chromosome 2 (2p21q). The most commonly used, inexpensive and widely available test is the lactose hydrogen breath test. This test aims to detect an abnormal increase in breath hydrogen excretion after an oral dose of lactose.

Unfortunately, there is no consensus on the optimal procedure to perform a lactose breath test, relating to administered dose of lactose (20–50 g of lactose), sampling interval (15, 30 or 60 min), test duration (2–5 h) and criteria for a positive test (10 or 20 ppm increase over baseline). Similarly, there is no consensus on whether it is recommended to measure CH_4_-excretion in breath in addition to H_2_.

The lactose breath test exhibits a high specificity (89%–97.6%) whereas a mean sensitivity of 77.5% has been reported [10,11]. Additional measurement of CH_4_-excretion has been proposed as a means to improve the accuracy of the test [12,13,14]. Indeed, generation of methane involves the reduction of CO_2_ by H_2_ to yield CH_4_ [15]. As this reaction removes considerable amounts of H_2_, it can result in a too low increase in H_2_ concentrations to result in a measurable increase in breath hydrogen and consequently yield a false negative result. These individuals are classified as H_2_–non-producers. In most studies, they account for <10% of the subjects [10].

In our hospital, we routinely apply a modified version of the lactose breath test by making use of stable isotope labelled (^13^C) lactose that allows to estimate the digestion of lactose in addition to the malabsorption of the sugar. Lactose that is digested in the small intestine results in the production of ^13^C-labelled glucose and galactose. The monosaccharides are absorbed and transported to the liver via the portal blood where they are oxidized to ^13^CO_2_ that is exhaled in breath. The test has been validated previously *versus* jejunal lactase activity [11].

This study was designed to evaluate the impact on the diagnostic accuracy of the lactose breath test when measuring breath CH_4_ in addition to H_2_ and ^13^CO_2_.

## 2. Experimental Section

### 2.1. Subjects

This retrospective study was performed at the Department of Laboratory Medicine of the University Hospitals Leuven (Leuven, Belgium). Data of all subjects that performed a combined ^13^C/H_2_ lactose breath test to examine lactose malabsorption between January 2014 and June 2014 were reviewed. Measurements of ^13^CO_2_, H_2_ and CH_4_, age, gender, Body Mass Index (BMI) for subjects older than 18 years, as well as symptoms recorded on the day of the breath test were extracted from the Hospital’s data management system and were processed.

The study protocol was approved by the Ethics Committee of the University of Leuven in accordance with the declaration of Helsinki (S58141).

### 2.2. Methods

#### 2.2.1. ^13^C/H_2_ Breath Test

The subjects performed a ^13^C/H_2_ lactose breath after an overnight fast as described previously [11]. Briefly, four baseline breath samples were collected in Exetainers^®^ (Labco Ltd., Ceredigion, UK) after which the subjects ingested 50 g (children <25 kg; 2g/kg body weight) of naturally enriched ^13^C-lactose (atom percent: 1.097%; Hanze Nutrition, Groningen, The Netherlands), dissolved in 250 mL of tap water. Subsequently, two breath samples were collected every 30 min for 4 h. During the test, subjects were not allowed to eat, drink or smoke. To keep CO_2_-production constant, physical activity was prohibited during the test and the subjects remained quietly seated. Patients were asked to report any discomfort experienced during the test or later on the test day.

#### 2.2.2. Analytical Methods

Breath H_2_-, CH_4_- and CO_2_-concentrations were quantified in a single run using a gas chromatograph (GC, Trace GC Ultra, Thermo Scientific, Pittsburgh, PA, USA) coupled to a thermal conductivity detector (TCD, Thermoscientific, Pittsburgh, PA, USA) and a flame ionization detector (FID, Thermo Scientific, Pittsburgh, PA, USA). One mL of breath was injected at a temperature of 90 °C with split ratio 1:20 and injector temperature of 110 °C. Chromatographic separation was achieved isothermally at 120 °C on a packed precolumn (Hayesep-N; 0.25 m; 80–100 mesh; 1/8”SS, Restek, Bellefonte, PA, USA) followed by a packed column (Carboxen 1000; 1.5m; 60–80 mesh; 1/8”SS, Restek, Bellefonte, PA, USA) and using nitrogen 5.0 as a carrier gas with a constant pressure of 96 kPa. H_2_ eluted from the column with a retention time of 0.8 min and was detected by the TCD with detector temperature at 290 °C, block temperature at 150 °C, transfer temperature at 140 °C, a reference nitrogen flow of 15 mL/min and a gain of 10. After 1 min, a heated valve (50 °C) switched the column effluent to a methanizer at a temperature of 350 °C to convert CO_2_ into CH_4_. Both CH_4_, eluting after 2.3 min and the converted CO_2_, eluting after 5.4 min, were detected by FID with a temperature at 250 °C, hydrogen flow at 70 mL/min and air flow at 350 mL/min. Data were processed using ChromQuest 5.0 (Thermo Scientific, Pittsburgh, PA, USA). Reference gas (Messer, Zwijndrecht, Belgium) containing 25.4 ppm H_2_, 2.8 ppm CH_4_ and 3.6% CO_2_ was injected every 20 samples for calibration and results were expressed in ppm for H_2_ and CH_4_ or in % for CO_2_. Breath samples that contained less than 1% CO_2_ were qualified as unreliable due to atmospheric contamination of alveolar breath samples and were excluded from further analysis.

The ^13^C-content in breath samples was analyzed using a continuous flow isotope ratio mass spectrometer (IRMS, ABCA, Sercon, Crewe, UK) and expressed as δ^13^PDB value. CO_2_-production rate was assumed to be 300 mmol/m² body surface area/hour for subjects older than 16 years or children with a body weight ≥80 kg. Body surface area was calculated using the weight-height formula of Haycock *et al.* [16]. For children <16 years and <80 kg, CO_2_ productions were calculated according to weight, age, and sex and based on published data about metabolic rates [17]. The measured delta values were converted to percentage of the administered dose of ^13^C excreted per hour and as cumulative percentage of administered ^13^C after 4 h. As our IRMS-system was linear for samples containing 0.6%–5% CO_2_ (*i.e.*, deviation of less than 0.6‰ from the δ^13^_CO2_ of completely filled exetainers with reference gas (3.6% CO_2_)), breath samples containing <0.6% CO_2_ were excluded from further analysis.

#### 2.2.3. Standard Interpretation of Breath Test Results

A cumulative excretion of 14.5% of administered ^13^C after 4 h was used as the cutoff value for discrimination between low and normal lactose digestion [11]. Increased H_2_-excretion was defined as an increase in H_2_ concentration >20 ppm above baseline levels.

Subjects were classified as normal lactose digesters when the cumulative ^13^C-excretion after 4 h exceeded 14.5% and no increased H_2_-excretion during the 4 h during test was observed. Subjects with a cumulative ^13^C-excretion after 4 h ≤14.5% were diagnosed as lactase deficient, irrespective of a concomitant increase in H_2_-excretion. A test result with a cumulative ^13^C-excretion >14.5% and increased H_2_-excretion, indicated contact of the administered lactose with bacteria despite normal lactase activity, either in the small bowel, suggesting small bowel bacterial overgrowth (SIBO), or in the large bowel, suggesting lactose malabsorption. As no information on transit is available, it is not possible to differentiate between both conditions.

#### 2.2.4. Additional Breath Test Parameters

Breath methane concentrations ≥5 ppm above baseline levels were considered as increased ethane production [12].

The time at which a significant increase in CH_4_ or H_2_ compared to baseline was observed in breath was defined as the time at which the CH_4_- or H_2_-excretion exceeded 2 SD (standard deviations) of all previous points above the running average of all previous points [18].

#### 2.2.5. Statistics

Statistical analysis was performed using SPSS 22.0 (SPSS Inc. Chicago, IL, USA). Normality was tested with the Shapiro-Wilks-test. When normality assumptions were not met, data were analyzed with non-parametric tests (Kruskal-Wallis (K-W) and Mann-Withney (M-W) test with Bonferonni correction), whereas normally distributed data were analyzed using One-way analysis of Variance (ANOVA) with post-hoc Tuckey test or an independent samples *t*-test.

Normally distributed data were shown as mean ± standard deviations (SD) whereas not-normally distributed data were expressed as median plus interquartile range (IQR). To investigate differences in the distribution of patients, a Chi-Square test was applied. The level for statistical significance was set at *p* <0.05.

Spearman correlation was used to evaluate correlation between BMI and CH_4_-production.

## 3. Results

### 3.1. Patient Characteristics

Between January 2014 and June 2014, 1355 breath tests were analysed in the University Hospital Leuven. Sixteen tests (1.2%) were excluded for variable reasons: one subject vomited shortly after intake of the ^13^C-lactose, two tests were stopped early, and 13 tests were not correctly executed. In addition, 288 subjects that had not reported whether they experienced any discomfort or not on the day of the breath test were excluded from further analysis. Finally, the results of 1051 breath tests were included in this analysis.

The subjects consisted of 313 men (30%) and 738 (70%) females. Of them, 178 were children, aged <18 years. The mean age was 36.2 ± 19.2 years and the mean body mass index amounted to 23.2 ± 5.1 kg/m², children excluded. Four percent of the adults were underweight and had a BMI < 18%, 59% had a normal BMI (18 ≤ BMI ≤ 25), 25% had overweight (25 < BMI ≤ 30) and 12% were obese (BMI>30).

**Figure 1:**
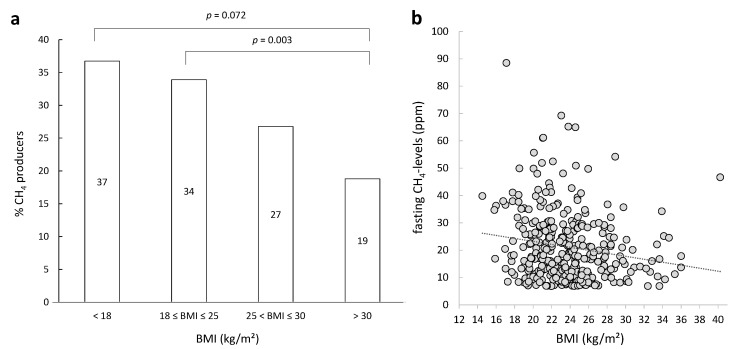
Percentage of CH_4_ producers in fasting state as function of body mass index (BMI) (**a**) and correlation between fasting CH_4_ and BMI (**b**).

Of the total cohort, 34% of the subjects had fasting levels of CH_4_ ≥5 ppm above baseline and 15% had fasting H_2_ levels >20 ppm. Significantly more patients with normal weight produced CH_4_ in fasting state compared to obese patients (*p* = 0.003). Similarly, more underweight than obese subjects produced CH_4_ (*p* = 0.072, Figure 1a). In addition, the extent of CH_4_ excretion was significantly negatively correlated to BMI within the group of subjects with baseline CH_4_ excretion >5 ppm (Spearman’s rho = −0.185, *p* = 0.000678). (Figure 1b).

### 3.2. Diagnosis

Of all the cases, 599 were diagnosed with normal lactose digestion (57.0%), 314 (29.9%) with lactase deficiency and 138 (13.1%) tests were suggestive of lactose malabsorption or SIBO.

Overall, 25% (265/1051) of the subjects produced H_2_ but no CH_4_, 12% (122/1051) produced CH_4_ but no H_2_, 3% (33/1051) produced both H_2_ and CH_4_, whereas 60% (631/1051) did not produce H_2_ nor CH_4_.

### 3.3. Occurrence of Symptoms on the Day of the Breath Test

Forty-one percent (435/1051) of all subjects did not experience any discomfort during or after the lactose breath test, whereas 59% (616/1051) reported one or more symptoms. Overall, 1216 symptoms were reported of which 93% were gastrointestinal complaints. Of these, cramps were present in 269 subjects (44%), flatulence in 240 subjects (39%), diarrhea in 196 subjects (32%), nausea in 104 subjects (17%), abdominal pain in 94 subjects (15%), bloating in 75 subjects (12%), eructations in 47 subjects (8%) and borborygmi in 40 subjects (6%). Besides gastrointestinal complaints, also systemic complaints were reported such as headache in 42/616 subjects (7%) and tiredness in 16/616 subjects (3%).

**Figure 2:**
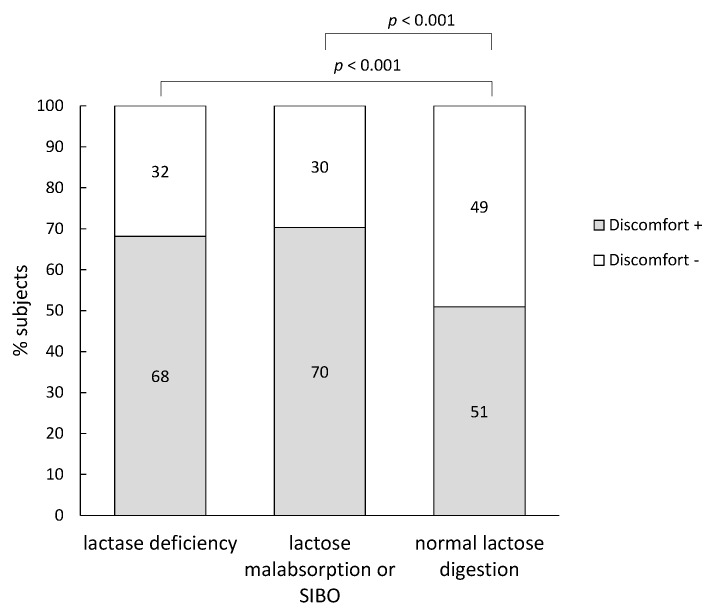
Percentage of subjects that reported discomfort after performing a ^13^C/H_2_ breath test.

Figure 2 shows that subjects with lactase deficiency and subjects with lactose malabsorption or SIBO reported significantly more discomfort than subjects with normal lactose digestion (*p* < 0.001 and *p* = 0.0009, respectively). Remarkably, also about half of the subjects with normal lactose digestion reported discomfort.

In addition, hydrogen production was significantly higher in those subjects (Table 1). Subjects with lactase deficiency that reported symptoms produced significantly more H_2_ than subjects without symptoms (*p* < 0.0001). Also in individuals with lactose malabsorption or SIBO, the H_2_-production was significantly higher when symptoms were present (*p* = 0.001). CH_4_-production was not related to the occurrence of symptoms.

**Table 1:** ^13^CO_2_, H_2_ and CH_4_ excretion according to diagnose.

Diagnosis	Number of Subjects	Cumulative ^13^C-Excretion after 4 h (%)	Maximum H_2_-Excretion (ppm)	Maximum CH_4_-Excretion (ppm)
Lactase deficiency	All subjects (314)	11.2 (8.4–13.0)	21.8 (3.7–96.5)	0.3 (0.1–1.7)
	Discomfort + (214)	10.7 (7.6–12.5) ^a^	60.9 (6.9–116.0) ^b^	0.3 (0.1–1.6)
	Discomfort − (100)	12.1 (9.4–13.7) ^a^	4.4 (1.8–17.6) ^b^	0.5 (0.1–2.0)
Lactose malabsorption/SIBO	All subjects (138)	19.0 (16.7–21.8)	48.1 (31.2–91.3)	0.2 (0.1–0.7)
	Discomfort + (97)	18.7 (16.5–21.4)	53.0 (34.3–103.0) ^c^	0.2 (0.1–0.8)
	Discomfort − (41)	20.1 (18.0–22.2)	37.0 (27.0–57.3) ^c^	0.2 (0.1–0.5)
Normal test	All subjects (599)	19.6 (17.4–33.2)	3.9 (1.5–9.5)	0.3 (0.1–2.4)
	Discomfort + (305)	20.2 (17.4–32.1)	3.5 (1.5–9.6)	0.3 (0.1–2.1)
	Discomfort − (294)	19.1 (17.2–33.2)	4.1 (1.4–9.1)	0.3 (0.1–2.5)

^a,b,c^ values with an identical subscript are significantly different (^a^ *p* = 0.00023; ^b^ *p* < 0.00001; ^c^ *p* = 0.001); values are median (IQR).

### 3.4. Subjects Diagnosed with Lactase Deficiency

A total of 314 subjects had a cumulative ^13^C-excretion <14.5% of administered ^13^C and were diagnosed with lactase deficiency. Remarkably, only 44% of these subjects were H_2_-producers (*n* = 139), whereas 8% were CH_4_-producers (*n* = 25), 7% produced H_2_ and CH_4_ (*n* = 21) and 41% were non-producers (*n* = 129). The extent of lactose digestion (cumulative ^13^C-excretion after 4 h) differed between the subgroups according to gas production (K-W, *p* < 0.001) (Table 2). The cumulative ^13^C-excretion was significantly lower in H_2_-producers compared to CH_4_-producers (M-W, *p* = 0.0004), the H_2_- and CH_4_-producers (*p* = 0.024) and the non-producers (*p* < 0.001).

Levels of H_2_-excretion in H_2_-producers were not different from that in H_2_- and CH_4_-producers (*p* = 0.952) and neither was CH_4_-production different in pure CH_4_-producers compared to H_2_- and CH_4_-producers (*p* = 0.316) (Table 2).

**Table 2:** ^13^CO_2_, H_2_ and CH_4_ excretion in patients with lactase deficiency.

Gas production	Number of Subjects	Cumulative ^13^C-Excretion after 4 h (%)	Maximum H_2_-Excretion (ppm)	Maximum CH_4_-Excretion (ppm)
H_2_-producers	All subjects (139)	9.4 (6.3–11.7)	95.5 (52.3–144.5)	0.2 (0.1–0.6)
	Discomfort + (117)	9.58 (6.0–12.7)	101.5 (64.7–148.3) ^a^	0.2 (0.1–0.5)
	Discomfort − (22)	8.8 (6.0–12.7)	50.7 (30.6–90.6) ^a^	0.3 (0.1–0.9)
CH_4_-producers	All subjects (25)	11.8 (11.1–13.4)	3.0 (1.0–9.2)	11.2 (6.8–15.6)
	Discomfort + (10)	11.6 (11.1–12.5)	3.2 (0.6–8.4)	11.3 (6.9–14.8)
	Discomfort − (15)	12.7 (10.9–13.5)	2.5 (1.6–8.2)	11.2 (7.4–19.8)
H_2_- and CH_4_-producers	All subjects (21)	12.4 (9.4–13.0)	94.5 (74.5–116.0)	14.8 (7.7–24.2)
	Discomfort + (21)	12.4 (9.4–13.1)	94.5 (74.5–116.0)	14.8 (7.7–24.2)
	Discomfort − (0)	-	-	-
Non-producers	All subjects (129)	12.2 (10.1–13.6)	3.7 (1.5–7.5)	0.2 (0.1–0.9)
	Discomfort + (66)	11.8 (9.7–13.1)	4.6 (1.5–8.1)	0.2 (0.1–0.6)
	Discomfort − (63)	12.5 (10.3–13.8)	3.0 (1.3–7.3)	0.3 (0.1–1.0)

^a^ significantly different (*p* = 0.002); values are median (IQR).

**Figure 3:**
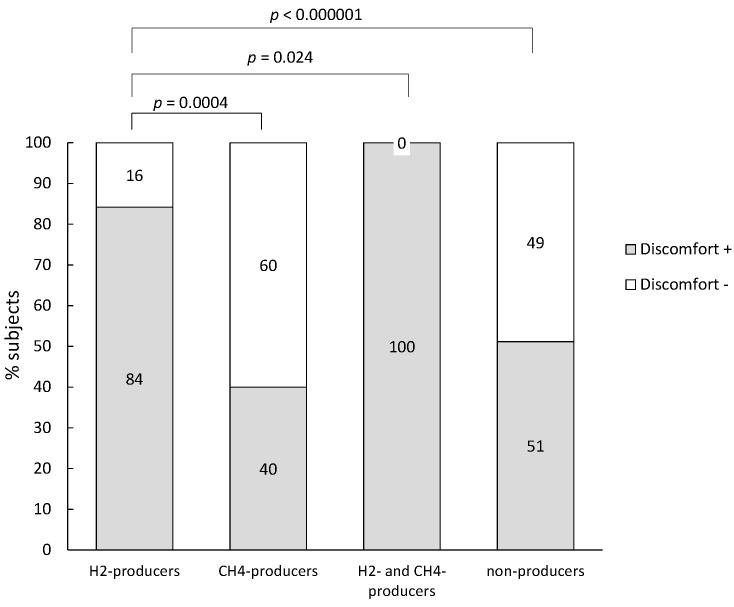
Percentage of subjects that reported discomfort within the subjects with lactase deficiency.

H_2_-producers reported significantly more discomfort compared to patients that produced no H_2_ (Chi Square, *p* < 0.001) (Figure 3).Within the group of subjects that only produced H_2_, H_2_-concentration was significantly higher for subjects that reported symptoms compared to subjects that did not experience symptoms (M-W, *p* = 0.002) (Table 2).

### 3.5. Subjects Diagnosed with Lactose Malabsorption or Bacterial Overgrowth

**Figure 4:**
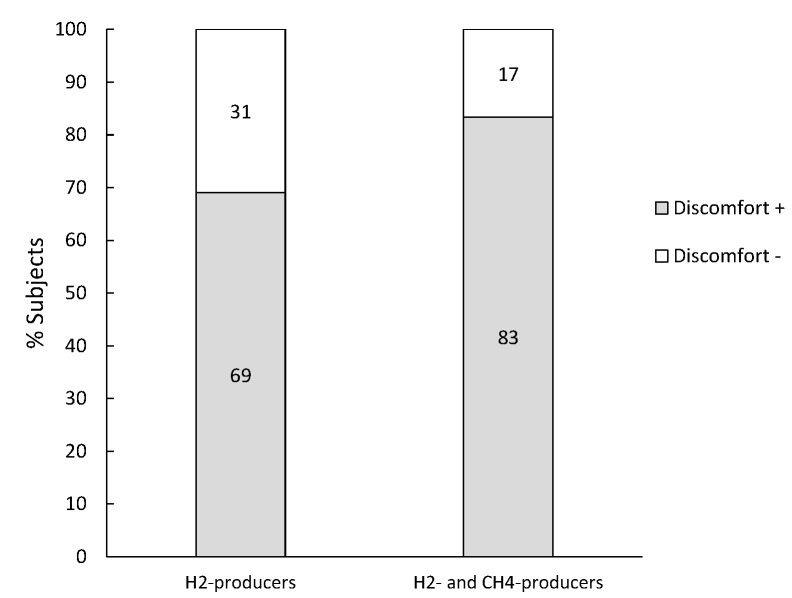
Percentage of subjects diagnosed with lactose malabsorption or SIBO that reported discomfort.

Subjects (*n* = 138) that produced at least 20 ppm H_2_ in combination with a normal lactose digestion were diagnosed with lactose malabsorption or SIBO. Only 12 of them (9%) produced both H_2_ and CH_4_ whereas the majority (126; 91%) produced only H_2_. No difference in percentage of patients that reported symptoms was observed between the H_2_-producers *vs*. H_2_- and CH_4_-producers (Figure 4).

Again, H_2_-excretion was significantly higher when symptoms were reported, both in the pure H_2_ producers (*p* = 0.003) as in the H_2_ and CH_4_-producers (*p* = 0.032) whereas complaints were not related to CH_4_-production (*p* = 0.470 and *p* = 0.273 for H_2_-producers and both H_2_- and CH_4_- producers, respectively) (Table 3).

**Table 3:** ^13^CO_2_, H_2_ and CH_4_ excretion in subjects diagnosed with lactose malabsorption/SIBO.

Gas production	Number of Subjects	Cumulative ^13^C-Excretion after 4 h (%)	Maximum H_2_-Excretion (ppm)	Maximum CH_4_-Excretion (ppm)
H_2_-producers	All subjects (126)	19.0 (16.7–21.6)	48.5 (31.2–91.3)	0.2 (0.1–0.4)
	Discomfort + (87)	18.3 (16.4–21.0) ^a^	57.7 (35.2–103.2) ^b^	0.2 (0.1–0.4)
	Discomfort − (39)	20.1 (18.1–22.1) ^a^	38.3 (27.5–57.6) ^b^	0.2 (0.1–0.4)
H_2_- and CH_4_-producers	All subjects (12)	20.8 (18.2–23.8)	41.0 (31.8–100.0)	20.9 (11.5–31.1)
	Discomfort + (10)	20.8 (19.1–24.0)	47.0 (35.1–100.0) ^c^	17.3 (10.5–29.9)
	Discomfort − (2)	19.3 (17.1–21.5)	25.0 (24.3–25.8) ^c^	46.8 (34.3–59.3)

^a,b,c^ values with an identical subscript are significantly different (^a^ *p* = 0.030; ^b^ *p* = 0.003; ^c^ *p* = 0.032); values are median (IQR).

### 3.6. Subjects Diagnosed with a Normal Lactose Digestion and H_2_ ≤ 20 ppm

**Figure 5:**
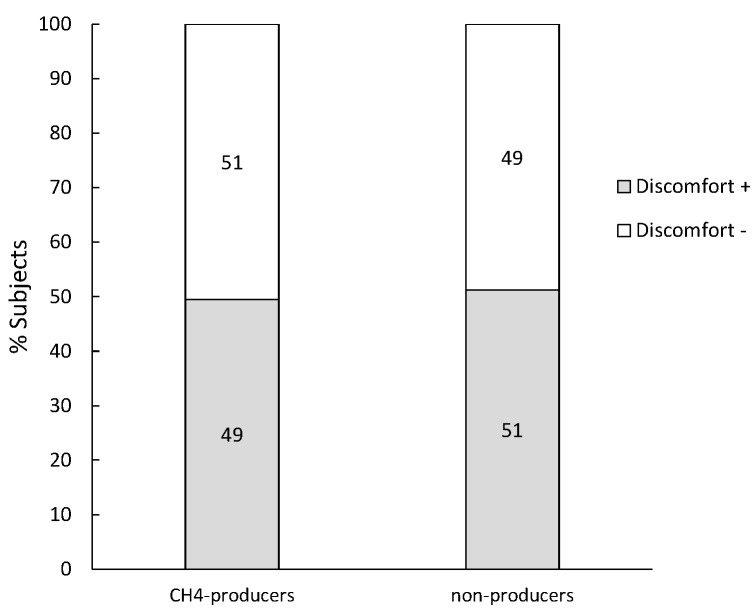
Percentage of subjects with normal lactose digestion that report discomfort.

Of the 599 subjects with normal lactose digestion, 97 (16%) produced CH_4_ (Table 4). Remarkably, about half of these subjects reported discomfort, irrespective of whether they produced methane or not (Figure 5).

**Table 4:** ^13^CO_2_, H_2_ and CH_4_ excretion in subjects with normal lactose digestion and H_2_ levels below 20 ppm.

Gas Production	Number of Subjects	Cumulative ^13^C-Excretion after 4 h (%)	Maximum H_2_-Excretion (ppm)	Maximum CH_4_-Excretion (ppm)
CH_4_-producers	All subjects (97)	19.2 (17.2–21.6)	3.5 (2.0–7.6)	10.2 (7.4–16.9)
	Discomfort + (48)	19.6 (17.2–21.7)	3.4 (2.0–7.7)	10.2 (7.8–16.6)
	Discomfort − (49)	18.9 (17.2–21.5)	4.7 (1.8–7.6)	10.2 (7.1–16.9)
non producers	All subjects (502)	19. 7 (17.4–22.4)	4.0 (1.5–9.5)	0.2 (0.1–0.6)
	Discomfort + (257)	20.3 (17.4–22.8)	4.0 (1.5–9.6)	0.2 (0.1–0.5)
	Discomfort − (245)	19.2 (17.5–21.9)	4.1 (1.3–9.2)	0.2 (0.1–0.8)

### 3.7. Increase in Methane *versus* Hydrogen in Breath

The cohort comprised 33 subjects that produced both H_2_ and CH_4_. H_2_-concentrations increased in breath at a slightly earlier time point (62 ± 40 min) than methane (67 ± 52 min) although this difference was not statistically significant (*p* = 0.786).

When comparing the raise in H_2_-production between CH_4_-producers (*n* = 41) and non CH_4_- producers (*n* = 320), no difference (*p* = 0.984) was observed (62 ± 40 min *versus* 65 ± 44 min).

### 3.8. Additional Value of CH_4_ Measurements

Addition of CH_4_ measurement to the current combined ^13^C/H_2_ lactose breath test yielded concordant results for 954/1051 (90.2%) tests. Discordant results were obtained in 97 patients with normal lactose digestion and no H_2_-production but with CH_4_-excretion in breath.

## 4. Discussion

Hydrogen breath testing is currently considered to be the diagnostic method of choice for the phenotypic detection of lactose malabsorption. It is a reliable, non-invasive and easy-to-perform test that offers the advantage over the genotypic test of including also secondary causes of lactose malabsorption. Nevertheless, false positive tests are possible in case of SIBO [19] whereas false negative tests might occur in methanogenic individuals that harbor a microbiota that avidly converts H_2_ into CH_4_. It is important to realize that the lactose breath test identifies lactose malabsorption rather than lactose intolerance and that not all individuals with lactose malabsorption experience discomfort. The mechanisms that provoke symptoms in some subjects and not in others are not well understood. Factors like fermentation characteristics of the microbiota [20], visceral hypersensitivity [21] and psychological factors [22] may be involved. Jellema *et al.* included the results of 18 studies that investigated the relationship between symptoms after lactose ingestion and the results of the lactose breath test in a systematic review and found that 33%–97% of the patients with a positive breath test reported symptoms [23]. In the present study, up to 68% of subjects diagnosed with lactase deficiency and a similar proportion of the individuals in which lactose malabsorption could not be differentiated from SIBO reported discomfort. Those subjects had significantly higher breath H_2_-excretion compared to the subjects without discomfort suggesting that high levels of gas production might contribute to symptom generation. Also, Hermans *et al.* found a strong correlation between gastrointestinal symptoms and extent of hydrogen excretion [24]. In a study in IBS (Irritable Bowel Syndrome) patients, both gas production and visceral hypersensitivity contributed to the development of digestive symptoms after oral lactose load, although hydrogen excretion was not associated with abdominal distention [25].

In addition, about half of the subjects with a normal lactose digestion reported discomfort. It is possible that the high dose of lactose used in this study (50 g), at least partially, explains the high rate of discomfort. As the combined ^13^C/H_2_ test was originally validated against jejunal lactase activity using 50 g of lactose [12], this dose was continued in the clinical routine. However, also other studies report that some individuals attribute abdominal symptoms erroneously to the intake of lactose and consider themselves as severely lactose intolerant although they are able to normally digest lactose [26]. Those symptoms may be due to other underlying disorders such as irritable bowel syndrome [27,28], or to a “nocebo effect” [29]. A nocebo effect is the effect of an inactive substance or procedure (placebo) suggesting that it will negatively modify a symptom or sensation. In those subjects, dietary measures and exclusion of lactose are unnecessary and will not improve the symptoms.

Symptom registration during a hydrogen breath test has been shown to have a good negative predictive value [30] as absence of symptoms excludes lactose intolerance. Also, lactose malabsorbers do not need further treatment in case they are lactose-tolerant. On the other hand, the diagnostic performance of the presence of symptoms (diarrhea, abdominal pain, bloating, flatulence and self-reported milk intolerance) was highly variable with positive predictive values ranging from 0.54–1.0 [23].

It is important to realize that the lactose breath tests performed in this study made use of ^13^C-lactose and estimated both the digestion of lactose (from exhaled ^13^CO_2_) and the malabsorption (from exhaled H_2_). The combined ^13^CO_2_/H_2_ breath test was more sensitive (0.84 *versus* 0.68) and more specific (0.96 *versus* 0.89) than the H_2_ breath test in detecting low jejunal lactase activity [12]. In the combined test, a diagnosis of low lactose digestion is based on a low cumulative ^13^CO_2_-excretion, irrespective of the presence of increased H_2_ which reduces the prevalence of false negative tests due to H_2_ non-producers. As a consequence, 154 subjects with low lactose digestion would have been classified as normal lactose absorbers on a standard H_2_ breath test. Twenty-five of them produced CH_4_ whereas the majority neither exhaled H_2_ nor CH_4_. Again, the prevalence of discomfort was higher in the subgroups that produced either H_2_ or H_2_ and CH_4_ compared to the subgroups that did not produce H_2_. The proportion of subjects that did not produce H_2_ nor CH_4_ despite a low lactose digestion was clearly higher that the proportion of non-H_2_-producers reported in most studies using the lactulose hydrogen breath test (2%–43% with <10% in most studies [11]). A potential explanation for this discrepancy might involve so-called colonic adaption. Several studies have indicated that breath H_2_-excretion decreases in subjects with lactose malabsorption after chronic consumption of lactose due to adaptation of the colonic microbiota [31,32,33,34]. Szilagyi *et al.* showed that the H_2_-output in a lactose breath test inversely varied with the lactose intake whereas there was no cross-adaptation to lactulose [35]. A radical decrease in lactose intake prior to the lactose hydrogen breath test was required to unmask lactose malabsorption. As a consequence, it is possible that a higher proportion of the population does not produce gas in a lactose breath test than in a lactulose breath test. An alternative explanation might be that the combined ^13^C/H_2_ lactose breath test in its current form is too sensitive and that subjects with a cumulative ^13^CO_2_ excretion below 14.5% that do not produce gas have a normal lactose digestion. However, when we reanalyzed our data using a cut off of 13.5% and 12.5% for normal lactose digestion, respectively, 37% and 35% of the subjects with low lactose digestion did not produce gas. This proportion remains far from the 10% proportion commonly found using a lactulose breath test.

Several studies have investigated the usefulness of combined measurement of H_2_ and CH_4_ to increase the accuracy of the H_2_ breath test. The rationale is based on the fact that methanogenesis is an efficient H_2_-consuming process that might reduce available H_2_ and prevent a rise in breath H_2_ by 20 ppm. Indeed, breath H_2_-production is significantly higher in subjects that do not excrete CH_4_ compared to CH_4_-excreters, both in the present study as in studies reported in literature [36,37]. However, the latest report of the Rome Consensus Conference published in 2009 does not recommend measuring breath CH_4_ excretion to improve diagnostic accuracy of the hydrogen breath test, due to insufficient evidence and conflicting results [11]. More recently, additional studies evaluating the usefulness of CH_4_ testing in addition to H_2_ observed better diagnostic properties for the combined H_2_ and CH_4_ test to detect lactose malabsorption [14,15,38]. Addition of CH_4_ measurement to the ^13^CO_2_/H_2_ breath test also further improved the accuracy of the test as 97/599 subjects with normal lactose digestion and no H_2_ excretion were found to excrete CH_4_. These subjects should have been classified as subjects with lactose malabsorption or SIBO. Nevertheless, the usefulness of breath CH_4_ measurements has been criticized as both false negative and false positive breath CH_4_ results are possible. McKay *et al.* showed that all healthy subjects may produce methane, but that only when the production reaches a threshold, it appears in the breath [39]. A recent studies in healthy subjects and IBS (Irritable Bowel Syndrome) patients showed that breath CH_4_-excretion is not a reliable marker for its colonic generation [40]. Methane was only detectable in breath when a significant amount was produced during colonic fermentation. In addition, some studies suggest that breath CH_4_-excretion is relatively stable during the day and is not responsive to changes in the diet because its generation depends on endogenous substrates although exogenous substrates like lactulose can significantly increase breath hydrogen [41]. False positive results may occur due to the release of CH_4_ entrapped in stool. Poor diffusion of gasses through the dense intestinal liquid allows the formation of gas bubbles that get entrapped along the colon where the content is becoming more solid [42]. It has been shown that in severely constipated patients, the entrapped gas can be released during a lactulose H_2_ breath test due to mixing of the intestinal content [43].

There is considerable evidence that methane production is positively associated with slow intestinal transit and constipation [44,45]. In addition, recent data from animal experiments supported by human observations [46] indicate a causal relationship between both conditions although the exact mechanism by which methane slows intestinal transit remains currently unknown [47]. To verify this observation, we investigated whether the orocecal transit time (OCTT), defined as the time that elapsed between intake of lactose and a rise in breath hydrogen excretion, was shorter in the subgroup of individuals that did not excrete CH_4_ compared to the CH_4_-excretors. In the present study, the OCTT was not different between both groups although a significantly shorter OCTT was found in non-CH_4_ excretors compared to CH_4_-excretors in a comparable study [37]. This discrepancy might be explained by the fact that our study was not designed to evaluate this observation and that the frequency of breath sampling (every 30 min) was too low to detect potential differences in OCTT.

Recently, some studies found an association between intestinal methane production and obesity with higher levels of methane in subjects with a higher BMI [48,49]. Within the subgroup of subjects that produced CH_4_ in response to a lactose challenge, there was no correlation between their BMI and the extent of CH_4_-production. Similarly, baseline levels of CH_4_ were not correlated to BMI in the complete study cohort (lactose absorbers and malabsorbers). In contrast, subjects that were underweight (BMI < 18) displayed even slightly higher CH_4_-levels compared to normal weight and obese subjects. This is in agreement with the fact that levels of *Methanobrevibacter smithii*, the dominant methanogen in the human intestine [16], were much higher in anorexic patients than in a lean population [50]. Similarly, a recent cross-sectional study that determined the association between fecal levels of Archaea (to which methanogens belong), methane production, fecal SCFA and BMI, concluded that colonic Archaea levels are not associated with obesity in healthy humans [41]. Two other studies found negative correlations between *M. smithii* levels and BMI [51,52]. Overall, the role of methane production in obesity remains inconclusive.

The cost of a combined ^13^C/H_2_ lactose breath test is higher than the cost of the standard H_2_ breath test due to the higher cost of the ^13^C-labelled substrate (700 €/kg). Due to the high capacity of the IRMS-system, allowing us to analyze up to 400 samples per 24 h (not limited to lactose breath tests), the cost of the additional analysis (including additional test tubes) is limited to €4.3. In Belgium, the social security system reimburses the cost of the breath test so that the additional contribution of the patient compared to a H_2_ breath test amounts to €9–11, depending on the insurance status of the patient.

## 5. Conclusions

This retrospective analysis confirms the poor correlation between abdominal discomfort reported during or after a lactose breath test and lactose malabsorption (32% of lactose malabsorbers did not report discomfort) or lactose intolerance (50% of normal lactose digesters reported discomfort). Measurement of ^13^CO_2_-excretion in addition to H_2_-excretion provides an added value to the standard hydrogen lactose breath test as it allowed detecting 154 non-hydrogen producers that would have been classified as normal lactose digesters using a standard test. Additional measurement of CH_4_ further improved the accuracy of the test and allowed identifying 97 methanogenic subjects with normal lactose digestion. Those subjects should have been classified as lactose malabsorption or SIBO patients.
